# Expression-Dependent Folding of Interphase Chromatin

**DOI:** 10.1371/journal.pone.0037525

**Published:** 2012-05-23

**Authors:** Hansjoerg Jerabek, Dieter W. Heermann

**Affiliations:** 1 Institute for Theoretical Physics, Heidelberg, Germany; 2 Interdisciplinary Center for Scientific Computing, Heidelberg, Germany; 3 The Jackson Laboratory, Bar Harbor, Maine, United States of America; 4 Shanghai Institute of Biological Sciences, Chinese Academy of Sciences, Shanghai, China; Newcastle University, United Kingdom

## Abstract

Multiple studies suggest that chromatin looping might play a crucial role in organizing eukaryotic genomes. To investigate the interplay between the conformation of interphase chromatin and its transcriptional activity, we include information from gene expression profiles into a polymer model for chromatin that incorporates genomic loops. By relating loop formation to transcriptional activity, we are able to generate chromosome conformations whose structural and topological properties are consistent with experimental data. The model particularly allows to reproduce the conformational variations that are known to occur between highly and lowly expressed chromatin regions. As previously observed in experiments, lowly expressed regions of the simulated polymers are much more compact. Due to the changes in loop formation, the distributions of chromatin loops are also expression-dependent and exhibit a steeper decay in highly active regions. As a results of entropic interaction between differently looped parts of the chromosome, we observe topological alterations leading to a preferential positioning of highly transcribed loci closer to the surface of the chromosome territory. Considering the diffusional behavior of the chromatin fibre, the simulations furthermore show that the higher the expression level of specific parts of the chromatin fibre is, the more dynamic they are. The results exhibit that variations of loop formation along the chromatin fibre, and the entropic changes that come along with it, do not only influence the structural parameters on the local scale, but also effect the global chromosome conformation and topology.

## Introduction

In the last decades the question how the genome of eukaryotic cells is organized during interphase has been subject to various studies. Known so far is that the organization of the genome is done on multiple length scales and levels of compactification, respectively. At the smallest scale, the DNA double helix in the cell nucleus is wrapped around histone molecules, forming a beads-on-a-string-like filament called chromatin with a diameter of about 11 nm [1]. In-vitro experiments have shown that this chromatin fiber can in turn condense to a filament with a diameter of 30 nm. However there is no proof for the existence of this filament in living cells and on the scale above 30 nm even less is known about how the genome is organized [2]. This lack of knowledge mainly arises from the difficulty of obtaining detailed structural information about chromatin in interphase. Experiments using light microscopes are limited in resolution and do not provide the required accuracy. Hence indirect experimental methods like fluorescense in-situ hybridization (FISH) and chromosome conformation capture (3C) are frequently used. While FISH experiments utilize fluorescent markers to determine the spatial position of selected genomic regions [3], [4], common 3C techniques like 4C, 5C and Hi-C [5]–[9] provide information about the spatial proximity of these regions.

A few years ago FISH studies were the first to reveal that the mean square spatial distance (MSD) between genomic regions that are separated by more than about 10 megabase pairs (Mb) remains almost constant [4]. As the diameter of the nucleus is only of the order of 10
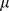
m, this so-called leveling-off is essential for making the whole genome fit into the nucleus. Modeling the chromatin fiber as a polymer chain, the observed characteristics of the MSD cannot be reproduced using any of the common polymer models, like the random walk (RW) or self-avoiding walk (SAW) [10]. Recently, the Fractal Globule (FG) model [11], [12] has become very popular as it is in agreement with results from Hi-C experiments [9]. Yet, the accordance with the data is only satisfied for a certain genomic range and as the model polymers do not exhibit a plateau in the MSD, the FG model does not allow to explain the folding of whole chromosomes.

Nonetheless, recent computational studies have shown that the leveling-off, and the related increased compaction of interphase chromosomes, can be explained by incorporating the formation of chromatin loops as a basic principle of genome organization [13]. While unlooped polymers would fill the whole nucleus, looped polymers condense and occupy volumes that are much smaller than the nuclear one. The same effect is observed for eukaryotic chromosomes which are confined within rather compact areas, their so-called chromosome territories [14]–[18]. On the one hand, this hints to the fact that chromatin looping contributes to the formation of the territories [19]–[21]. On the other hand, the loops additionally provide a good explanation why the chromosomes segregate in interphase nuclei, namely because loops effectively repel each other [22]. This entropic repulsion depends on the number and size of loops and prevents too strong intermingling of chromosomes. This effect can even be observed for mutually non-permeable polymers without excluded volume [23]. Preventing full intermingling of chromosomes is especially important for the organization of eukaryotic nuclei because strongly interspersing chromosomes would complicate cell division as the multiple chromatin fibres would have to be disentangled during mitosis or meiosis.

While some chromatin models expect only loops of certain sizes to be formed [24], 3C-like experiments provide evidence that loops of all sizes can be found in-vivo and that chromatin contacts can be established even between loci that are genomically seperated by hundreds of Mb. However, even though it has been shown that the leveling-off as well as the formation of chromosome territories can theoretically be explained with random non-specific looping [4], [13], [25], chromatin loops have also been reported to play a role in gene regulation. They can alter gene transcription by forming functional contacts between genes and regulatory genomic elements like enhancers or silencers [26], [27]. These associations correlate with an increase or reduction of the expression level of certain genes. For the beta-globin locus, for example, experiments have demonstrated that these functional contacts are indeed able to influence transcription [28].

The mentioned results suggest that chromatin looping could be one of the main driving forces behind genome organization and transcriptional regulation. As looping largely influences the folding of the fibre, genomic regions exhibiting different levels of expression can be expected to exhibit structural and topological differences. Recent FISH studies support this assumption because they show that the MSD between genomic loci is larger in transcriptionally highly active regions than in less active ones [4]. Furthermore, there is evidence that chromatin density is higher in lowly than in highly expressed regions [3]. Additionally to this structural variations, multiple studies have shown that chromosomes also exhibit an expression-dependent topology, with frequently transcribed genes being preferentially located at the periphery of their chromosome territories and the lowly expressed loci being situated closer to the center [16], [29]–[31]. However, there are also studies stating that the periphery is on average as transcriptionally active as the whole chromosome territory [32] and that transcription sites are basically uniformly distributed throughout the nucleoplasm and the territories [33], [34].

In this computational study we use a recently established loop formation model to relate transcriptional activity to structural properties of chromatin. We implement information from gene expression profiles into the Dynamic Loop (DL) model [13] to generate possible chromosome conformations. To verify the consistency of our model, we compare the structural and topological parameters of the simulated fiber to recent results from FISH and Hi-C experiments [3], [4], [9].

## Results

The results presented here are obtained by calculating the average of the respective observables over thousands of uncorrelated chromosome conformations, which corresponds to averaging over a large number of cells. Hence, our conclusions always refer to the ensemble of possible conformations, which does not mean that every single conformation would allow to derive the exact same conclusions. The structural and topological variations between the single conformations can be seen to reflect the cell-to-cell variations.

### Relation between Spatial and Genomic Distance

**Figure 1:**
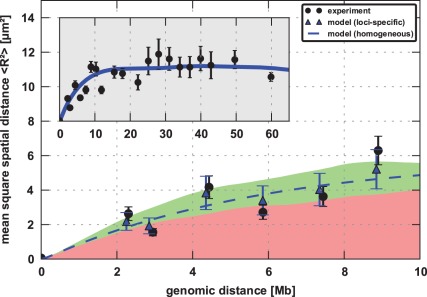
Mean square spatial distance (MSD) in chromosome 11 for human female primary fibroblast (04–147): The obtained average MSD for the total fibre (inset, blue line) is in good agreement with experimental data [**4**] (inset, black circles). The loci-specific MSD calculated from the simulated conformations (blue triangles) shows the same fluctuations between highly and lowly active loci as the experiment does (black circles). The MSD calculated for a fibre with homogeneous affinity (dashed blue line), meaning without expression dependence, is between the MSD for highly and lowly active regions. The MSD calculated specifically for highly transcribed regions (green area) exhibits a larger MSD than regions with less expressed loci (red area) because they are less compact. The corresponding regions taken for the analysis are highlighted in green and red, respectively, in Fig. 2 and were taken from [3].

To obtain information about the average spatial distance that genomic loci are seperated by, we used the generated chromatin conformations to calculate the mean square spatial distance (MSD) between them. This quantity is also calculated in the FISH experiments we compare our results to [4]. However, since the FISH measurements only provide information about the physical distance between specific genomic loci and not for the whole genome, we determined the average MSD for the total fibre (MSD*_total_*) as well as between the monomers that represent the FISH loci (MSD*_FISH_*) (see Materials and Methods). In Fig. 1 one can see that there is a good agreement between the FISH data and the obtained results. The simulations with the DL model allow to consistently reproduce the leveling-off above genomic distances of ≈10 Mb. Like in the experiments, the obtained MSDs show that there is an expression-dependent difference in the degree of leveling-off. The lowly expressed regions exhibit a MSD lower than the average and hence are more compact. In contrast to this, an increased MSD and a reduced leveling-off can be found in frequently transcribed parts of the fibre, indicating that these regions are less dense.

**Figure 2:**
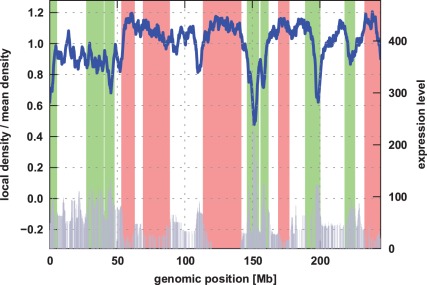
Local chromatin density: The fibre density around frequently transcribed loci (green) is smaller than in lowly expressed regions (red). Thus, on average, they do occupy a larger volume, indicating an increased decompactification of the highly transcribed parts. The bars (gray) represent the expression profiles and the green and red areas mark highly and lowly active regions, respectively [3].

As some experimental FISH markers bind to transcriptionally highly active regions while others bind to less active ones, the experimental datapoints fluctuate around the MSD*_total_* obtained from the simulation because it only represents the average MSD of the fibre. Thus we additionally compared the calculated loci-specific MSD*_FISH_* with the given data. As one can see in Fig. 1, the calculated MSD*_FISH_* shows the same expression-dependent fluctuations as the corresponding experimental data, with the lowly active loci being much closer to each other in space. This means that the model can reproduce the average fibre behavior as well as the loci-specific variations that result from the expression-related heterogeneity of the chromatin fibre.

### Local Chromatin Density and Volume Ratio

In order to verify that less active regions are more compact than highly active ones, we calculated the three-dimensional Gaussian kernel density estimation [35] for the simulated chromosomes. This method allows to determine the local chromatin density and thus is measure for the local chromatin compactness. In Fig. 2 one can see that the parts of the polymer that represent highly expressed loci exhibit low density, while less active regions are much more dense. The correlation coefficients between the expression profiles and the chromatin density are between −0.55 and −0.70 for both Pearson and Spearman correlation (see Tab. 1) [35]. As the p-values are very small (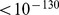), we can confirm the significant anticorrelation between expression and chromatin density. Hence, the model can successfully reproduce the experimentally observed expression-dependent variations in chromatin compactness [3]. However, the local chromatin density is just a qualitative measure. To also get a quantitative comparison, we calculated the dimensionless volume ratios 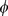 between highly and lowly active parts (see Materials and Methods) for the same regions that were investigated in a recent experimental study [3]. The average volume ratios obtained from the generated conformations vary between 1.39 and 1.52 for the different chromosomes (see Tab. 1). This is close to the experimental result, where an average volume ratio of ≈1.4 has been determined [3].

**Table 1:** Averages of structural parameters for chromosomes 1 and 11 for human female primary fibroblast (04–147) and K562 lymphoblastoid.

Chromosome (cell type)	1 (147)	1 (K562)	11 (147)	11 (K562)
Lattice scaling [ 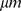 ]	0.073	0.076	0.075	0.076
Number of loops/length of polymer [%]	40.2	42.3	41.9	40.8
Volume ratio (highly/lowly expressed)	1.52	1.43	1.39	1.48
Loop exponent 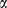 (total chromosome)	−1.18	−1.12	−1.19	−1.16
Loop exponent 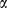 (highly expressed regions)	−1.26	−1.31	−1.30	−1.31
Loop exponent 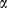 (lowly expressed regions)	−1.04	−1.08	−1.10	−1.09
Asphericity (total chromosome)	0.18	0.14	0.15	0.16
Asphericity (highly expressed regions)	0.38	0.34	0.33	0.34
Asphericity (lowly expressed regions)	0.25	0.26	0.23	0.21
Pearson correlation between local				
chromatin density and expression profile	−0.70	−0.64	−0.69	−0.69
Spearman correlation between local				
chromatin density and expression profile	−0.60	−0.58	−0.61	−0.55
Pearson correlation between squared				
distance to center and expression profile	0.72	0.65	0.69	0.71
Spearman correlation between squared				
distance to center and expression profile	0.59	0.56	0.60	0.55
Pearson correlation between squared				
distance to surface and expression profile	−0.66	−0.65	−0.64	−0.61
Spearman correlation between squared				
distance to surface and expression profile	−0.57	−0.56	−0.60	−0.56

### Chromatin Looping

**Figure 3:**
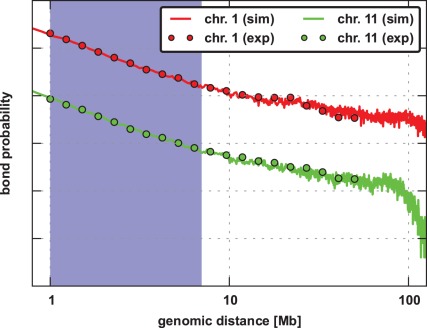
Log-log plot of loop size distributions for chromosomes 1 and 11 of K562 lymphoblastoid (continuous line) with the corresponding data from Hi-C experiments (circles) [**9**]. As the distributions for the chromosomes are strongly overlapping, the graphs were shifted by different constants in order to improve the illustration of each distribution.

Previous results from Hi-C experiments [9] pointed out that chromatin loop distributions for loop lengths g between ≈0.5–7 Mb are proportional to 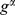, with 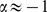 (see sec_methods). For the generated conformations we calculated the loop distributions for the total fibre and determined 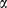. The results presented in Fig. 3 and Tab. 1 clearly show that the obtained contact distributions agree well with the experimental data [9]. Due to the coarse-graining used here, with one monomer equaling 150 kb, the model can not make any predictions for scales below 0.5 Mb. However, the model can reproduce the general loop formation behavior of real chromatin fibres on the scale between 0.5–7 Mb but also for longer genomic distances where the distributions are no longer proportional to 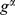.

As we are here interested in the differences between highly and lowly expressed regions, we furthermore determined the loop distributions specifically for monomers in these regions. In Tab. 1 one can see that the loop distributions show an expression-dependence: For highly active loci the exponent 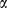 is more negative, leading to a faster decrease of the distribution for short loops between 0.5 and 7 Mb. The differences in behavoir probably arise from the variation in compaction: As the highly transcribed regions are less dense, the loci are on average separated by larger spatial distances, making the chromatin-chromatin interaction less probable.

### Shape of Chromosomal Regions

As we have seen that the volume of different parts of the fibre depends on the local expression, we wanted to know whether these parts also vary in shape, i.e. if they are spherical or more ellipsoidal. Using the simulated chromatin conformations, we determined the asphericities for the whole chromosomes as well as for the corresponding highly and lowly transcribed regions (see Materials and Methods). The results in Tab. 1 show that the total chromosomes are only slightly ellipsoidal with asphericities between 0.14 and 0.18. This is in disagreement with other computational and experimental studies that observed less spherical shapes [21], [36]. Real chromosomes within spherical mouse pro-B nuclei for example have been found to have an ellipsoidal shape with a ratio of principle axes of 1∶2.9∶4.5. This corresponds to an asphericity of ≈0.32. The observed difference results from the fact that we simulated single chromosomes that are not surround by others. For subparts of the fibre, which are surrounded by other subparts, comparable to chromosomes being encircled by other chromosomes, this effect is strongly reduced. For the generated conformations for example, the average asphericity of genomic regions with a length of 10 Mb is ≈0.28 which is much closer to the experimental values.

As far as the expression-dependence is concerned, the simulations show that the volume asphericity of highly expressed regions is much larger than of less active ones. While the asphericity is between 0.21 and 0.26 for lowly active, it is between 0.33 and 0.38 for highly active regions. Hence, the highly expressed parts of the chromatin fibre are more ellipsoidal. This is in agreement with experimental results on active and inactive chromosome X which have found that the inactive chromosome has a much more spherical shape than its active counterpart [37]. As a sphere has the smallest surface area among all surfaces enclosing a given volume, we can conclude that the less spherical highly expressed regions of the simulated polymers have a larger surface area and consequently provide more possible interaction sites, for example for the binding of proteins and protein complexes.

### Relative Position of Loci Inside their Chromosome Territories

**Figure 4:**
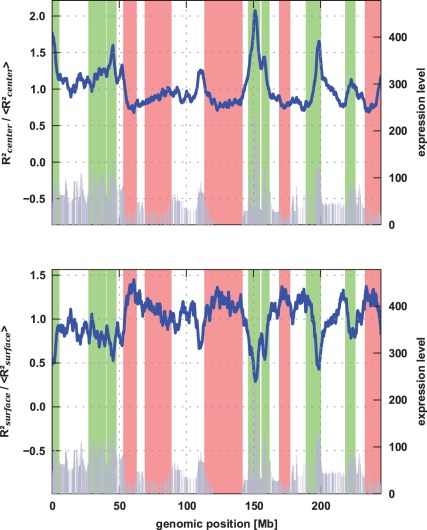
The mean square distance of genomic loci to the center-of-mass of the chromosome territory (top, blue line) depends on the corresponding expression level [**3**] (gray): The lowly transcribed regions are closer to the center than more active parts of the fibre. The mean square distance to the surface of the chromosome territory (bottom, blue line) confirms that the highly expressed loci preferentially reside closer to the surface of each territory than lowly expressed regions.

To obtain information about the relative position of specific loci inside a chromosome territory, we calculated the average distance to the center-of-mass (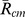) and to the surface (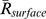) of the territory, respectively. The results exhibited in Fig. 4 clearly show that these distances strongly depend on the expression levels of the genomic regions the monomers represent. For the correlation between the expression profiles and the squared distance to the center, high Pearson correlation coefficients between 0.65 and 0.72 and Spearman correlation coefficients between 0.55 and 0.60 were obtained (see Tab. 1) [35]. Negligible p-values (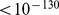) confirm the significance of the correlation. For the squared distance to the surface, an anticorrelation with the same degree of significance is observed. The lowly transcribed loci are found to preferentially locate closer to the center of the chromosome territory, while the highly active regions reside much closer to its surface. This topological property of chromosome territories has been stated by various experimental studies [29], [38]–[40]. Our results do not lead to the conclusion that the highly expressed regions must always be located at the surface since we also find chromosome conformations with highly transcribed regions being located close the center. Yet, on average these regions are more likely to be found at the periphery of the territories.

The observed expression-dependent positioning is expected to effect the connections between different chromosomes in two ways: On the one hand, chromosome intermingling is increased because the entropic loop repulsion is reduced due to the fact that the highly expressed regions close to the surface form less intrachromosomal loops [19], [22]. On the other hand, as the intermingling between chromosomes mainly occurs between genomic regions close to the surface of the territory, we can deduce that active regions of neighboring chromosomes are more likely to intermingle than the inactive ones which prefertially reside near the territory center. These effects are in agreement with recent studies where extensive chromosome intermingling as well as transcription-dependent interchromosomal associations have been observed in mammalian cells [32].

The obtained expression-dependent topological organization furthermore suggests a mechanism to make transcription more effective: When transcription more frequently takes place in the periphery of each chromosome, intermingling territories can share transcription factories [41]–[43], i.e. protein complexes that are expected to be responsible for transcription. Thus, in order to reach the same level of transcritpional activity, less factories would be needed than in the case of a totally random organization. In this context, a recent study has shown that transcription factories can frequently be found in intermingling chromosomal regions [32]. Studies where transcription sites were found to be mainly uniformly distributed throughout the nucleus [33], [34] are not necessarily in contradiction to our results since in some of the generated chromosome conformations highly active regions can also be found closer to the center of the corresponding chromosome territories. However, the ensemble average clearly exhibits the presented expression dependence.

### Mobility of Loci in their Chromosome Territories

**Figure 5:**
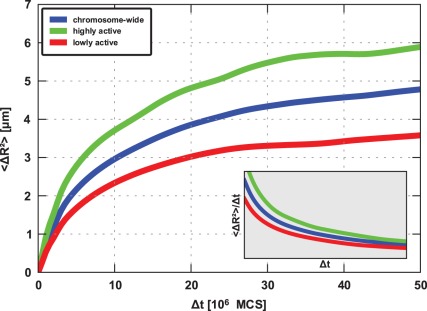
Mean square monomer displacement (MSMD) averaged over highly and lowly active regions on chromsomes 1 and 11: Highly expressed loci (green) are on average more dynamic than their less active counterparts (red). The positions of the regions were taken from [3] and are highlighted in Fig. 2. As the parts located at the ends of the polymer exhibit different diffusional behavior due to edge effects, they were excluded from the analysis. The blue curve represents the average MSMD over the total fibre. The insets (gray) show the MSMD divided by 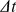.

In order to investigate the mobility of loci inside their associated chromosome territories, we calculated the mean square monomer displacement (MSMD) for each part of the polymer chain. It provides information on how much distance a monomer covers inside its territory, i.e. relative to the center-of-mass of the polymer, in a given timeframe. Hence, the MSMD allows conclusions about the movement of certain parts of the chromatin fibre inside its chromosome territory [15]. As one can see in Fig. 5, the MSMD depends on the expression of the genomic region under consideration: For highly transcribed regions the MSMD increases much faster than for lowly expressed ones, indicating that frequently transcribed parts of the chromatin fibre are much more dynamic than the less active parts, which appear to be rather static. This is in agreement with studies suggesting that the formation of large transcription factories hints to the possibility that during transcription the chromatin fibres rather than the RNA polymerases are the mobile partners [43], [44].

The insets in Fig. 5 show the MSMD divided by 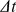, which should be constant in case of normal diffusion. As this is not the case, the monomers exhibit an anomalous diffusion, which arises from the fact that they are confined inside their respective chromosome territory.

## Discussion

The obtained results clearly show that we can generate chromatin conformations whose structural and topological properties are qualitatively as well as quantitatively in good agreement with experimental data. By making loop formation depend on transcriptional activity, the model allows to consistently reproduce multiple features of interphase chromosomes. We find that heterogeneous looping strongly influences the local structural parameters, like for example the MSD and chromatin density. Yet, for the simulated chromatin fibres we observe that the changes are not just limited to the local scale. They also effect the conformation and topology of the whole chromosome, meaning on the global scale. In this context, the main topological property, namely the relative position of selected genomic regions inside a chromosome territory, exhibits that the preferential location of given chromosomal regions depends on the corresponding transcriptional activity: The lowly expressed loci are on average located closer to the center, while the highly active ones tend to reside closer to the surface of the related territory. Even though this holds true for the average over the generated chromosomes conformations, it need not be true for each of them. However, it suggests that this kind of expression-dependent topology could make transcription more efficient because neighboring chromosomes could share transcritpion factories. Furthermore, the results for the mean square monomer displacement have shown that the highly transcribed regions on the outside of each territory are much more dynamic. Since this makes structural changes in these regions more likely, the increased dynamics could alter gene expression by for example modifying the function of regulatory elements: The more dynamic the fibre is, the sooner an enhancer or silencer can be expected to find the gene it regulates. Additionally the observed expression-dependent dynamics support the idea that not the transcription factories, but the chromatin could be the mobile partner during transcription.

## Materials and Methods

### Chromatin Model

The chromatin model used for the simulations was the Dynamic Loop (DL) model [13] where the chromatin fiber is represented by a polymer chain. As human chromosomes have lengths of the order of 100 Mb, detailed simulations at the level of single base pairs would be too time- and memory-consuming. Hence the freely jointed polymer is a coarse-grained version of the chromatin fiber where each monomer represents a long stretch of DNA. Such an approach is legitimate if the persistence length 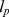 of the fiber is much smaller than the size of the stretch because then the polymer is expected to be totally flexible. Assuming a persistence length below 250 nm [45] and a fiber packing, where a length of 10 nm 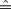 1 kilo base pair (kb), the monomers should represent DNA stretches with a length of at least 50 kb.

In the model the formation of chromatin loops is allowed and achieved as follows: Whenever two monomers get into spatial proximity due to diffusional motion, there is a certain probability that these two sites remain co-localized. In case a contact is created, a lifetime 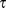 (chosen from a Poisson distribution) is assigned to it, determining when the bond dissociates. In this way the loop distribution along the polymer chain is dynamic and changes during the course of time, imitating the effects of temporary DNA-DNA interactions. Since monomers can only bind to each other if they are in close spatial proximity, the model does not introduce any kind of long range interaction between the different parts of the fibre. Due to the probabilistic creation of chromatin contacts it is not necessary to explicitly incorporate real interaction potentials into the model. Nevertheless, interaction affinities have to be assigned to each monomer as a measure for the probability to establish chromatin bonds. In general, an increase of this probability has two effects: On the one hand, as more bonds are formed the local structure becomes more compact because bonded monomers are hindered from diffusing apart. On the other hand, the loops that are established due to the bond formation effectively repel each other and prevent local intermingling of the fibre [19]. Due to this entropic effect, the high affinity can lead to a decompaction.

In this study the interaction affinities of the monomers are calculated from the expression profiles of the corresponding chromosomes in order to investigate whether the expression level is a good indicator for the properties of the chromatin fibre and whether the obtained activity-dependent structural changes are in agreement with experiments. Here, a low affinity is assigned to monomers representing highly expressed loci and the affinities of the chain increase with decreasing transcriptional activity. This is done because in highly active parts of the chromatin fibre much more proteins are present in order to facilitate transcription. Due to this, direct DNA-DNA interactions are less likely as it is more probable that proteins can be found between the fibres. Consequently, in our model the minimum interaction affinity 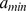 is assigned to the region exhibiting the highest expression level, while the maximum affinity 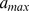 is assigned to regions that are not expressed. In the simulations the interaction affinities of the i*^th^* monomer are determined by
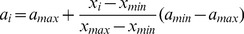
(1)with 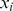 being the expression level at monomer i and 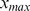 and 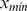 being the minimum and maximum expression value, respectively. From the affinities, the looping probability between the i*^th^* and j*^th^* monomer is then given by



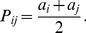
(2)As mentioned before, 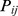 is only larger than zero if the distance between the i*^th^* and j^th^ monomer is lower than a given cutoff distance, which has been set to be equal to the maximum bond length between adjacent monomers along the chain. Hence, two non-adjacent monomers have to be spatially closer to each other than their neighbors along the chain to allow the formation of a bond. This means that the model incorporates only short range interactions and does not force parts of the chromsomes that are far away from each other in space and/or along the chain to come together.

For the generation of chromatin conformations Monte-Carlo simulations [46] were performed on a cubic lattice with periodic boundary conditions. The algorithm used is the well-tested bond fluctuation model [17], [48]. Each monomer of the chain occupies one lattice site, meaning that the monomers cannot co-localize. In order to prevent interactions between regular monomers and periodic images of monomers, the linear lattice size L must be chosen large enough, i.e. L must be much larger than the polymer’s radius of gyration. Otherwise biased conformations would be produced due to periodic backfolding. During the simulation the bonds along the backbone of the polymer are fixed and cannot break open. This means that DNA strand breaking, for example facilitated by topoisomerases, is not incorporated. However, as we simulate coarse-grained chromatin fibres, structural changes related to DNA breaking and the subsequent unwinding take place on a scale far below what is represented as a single monomer in the simulations.

In each Monte-Carlo trial move a monomer of the chain is randomly selected and, if possible, randomly moved to one of its nearest neighbors on the cubic lattice. Excluded volume interactions are taken into account by preventing a lattice site to be occupied by more than one monomer. When simulating a polymer chain of length N we define one Monte-Carlo step (MCS) to be equal to N trial moves. Hence each monomer is on average translated once during each MCS. As the monomers are only moved locally, subsequent conformations do not show significant structural differences. To obtain uncorrelated chromatin conformations for our analyses, we calculate the autocorrelation function 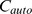 of the squared radius of gyration 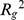
(t). Since
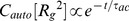
(3)we determine the estimated autocorrelation time 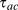 using an exponential fit. After 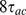 MCS two conformations are expected to be independent.

### Simulation Setup

In the simulations each monomer represents a DNA segment with a contour length of 150 kb. For comparison with experimental results we performed simulations of chromosomes 1 and 11 which were represented by polymers with chain lengths of N = 899 and N = 1634. The lattice size was set to L = 600 to avoid interactions caused by periodic boundary conditions. The mean of the Poisson distribution that is used to determine the bond lifetime 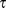 was set to 8000 MCS. The minimum interaction affinity of the monomers was set to 0.01, while the maximum affinity was set such that the best agreement, meaning the smallest average deviation between in-silico and in-vivo mean square distance was obtained. For both chromosomes 1 and 11 and both cell types under investigation, a maximum affinity between 0.06 and 0.07 was found to reproduce the FISH data best. The expression levels for the derivation of the polymers’ interaction profiles were provided by the lab of Roel van Driel (University of Amsterdam, Science Faculty, SILS: Nuclear organization Group) and are also available at NCBI (www.ncbi.nlm.nih.gov/geo) under the GEO accession no. GSE6890 and GSM153780 [3]. The expression data used are those of human female primary fibroblast (04–147) and K562 lymphoblastoid, respectively. These two cell types were chosen because the FISH measurements we compare the in-silico mean square distance to were carried out with female primary fibroblast (04–147) and because the distributions of intrachromosomal contacts are available from Hi-C experiments on K562 lymphoblastoid. As one can see in [3], the expression profiles have similar characteristic features, like position and width of highly and lowly expressed regions and height and position of expression level peaks etc. Due to the coarse-graining in our simulations, with one monomer being equal to 150 kb of genomic content, the cell-type specific changes in expression level and subsequently in looping probability are small compared to the differences between highly and lowly expressed regions. Hence, this study focuses on expression-dependent structural variations.

After determining the interaction profiles of the polymers, each simulation starts with an equilibration run of 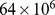 MCS where the formation of loops is rejected, leading to a random starting conformation of the polymer chain. Then a second equilibration run of again 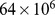 MCS is performed where the establishment of loops is allowed. In the subsequent main simulation run the chromatin conformations are saved every 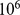 MCS, until at least 1000 independent conformation are generated for each chromosome.

### Mean Square Distance

The mean square distance (MSD) provides the average spatial distance that two monomers or loci are separated by as a function of the contour length or genomic distance between them, respectively. With 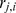 being the coordinate vector of the i*^th^* monomer of the j*^th^* conformation and n being the contour length along the polymer chain, the MSD is calculated as follows:
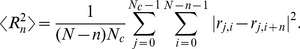
(4)


Here, N is the number of monomers the polymer consists of and N*_c_* is the number of available polymer conformations. As the simulations are carried out on a lattice, the coordinates 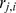 and consequently the MSD are given in lattice units. The scaling factor that relates lattice units to real units is obtained by scaling the computed to the experimental MSD (scaling factors see Tab. 1).

From FISH data the MSD cannot be calculated as in Eq. 4 because not all the coordinate vectors 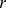 are known. Instead, the MSD is only calculated for certain pairs of loci, namely those that have been marked fluorescently. Hence, in order to correctly compare experiment and simulation, we also have to determine the loci-specific MSD for the generated chromosome conformations
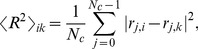
(5)with i and k being the monomers that correspond to the loci that have been marked in the FISH experiment.

### Loop Distribution

The loop distribution 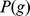 provides information about how probable it is that a loop of length g is formed. In general, this probability is proportional to the maximum number of loops that can possibly be established, namely
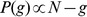
(6)with N being the total length of the polymer chain. For short loops with a genomic length between 0.5 and 7 Mb, experiments on human cells [9] have shown that the probability is furthermore proportional to:



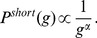
(7)From the chromatin conformations generated we can determine the loop distribution for the total fibre as well as for specific regions. The exponent 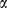 for short loops is then determined from the slope of 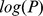 as a function of 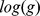 (see Fig. 3).

### Volume Ratio

The volume ratios of different genomic regions are calculated from the corresponding monomer densities of the simulated polymer chains. These densities are obtained using three-dimensional Gaussian kernel density estimation (GKDE) [35]. The volume ratios then are indirectly proportional to the density ratios:
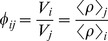
(8)


Here, 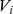 is the volume and 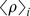 is the average density of all monomers in the i*^th^* genomic region.

### Volume Asphericity

To calculate the asphericity of the volume a polymer occupies, the gyration tensor **Q** of the monomers has to be determined [49]. The elements of the tensor are given by
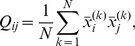
(9)where 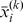 is the i*^th^* coordinate of the center-of-mass position of the k*^th^* monomer. Calculating the eigenvalues 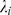 of the gyration tensor, the asphericity is
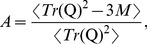
(10)with



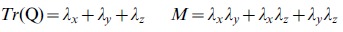
(11)The normalization is chosen such that the asphericity is 0 if the spatial monomer distribution is totally spherical and 1 for rod-like structures.
